# Psychosocial factors associated with intention to pursue tertiary education among Malawian students: the moderating effect of mental health

**DOI:** 10.1186/s40359-024-01562-7

**Published:** 2024-02-09

**Authors:** Jurgita Slekiene, Kondwani Chidziwisano, Elizabeth Tilley

**Affiliations:** 1 Global Health Engineering (GHE), Department of Mechanical and Process Engineering (D- MAVT), ETH Zurich, Clausiusstrasse 37, Zurich, 8092 Switzerland; 2 Department of Consultation-Liaison Psychiatry and Psychosomatic Medicine, University Hospital Zurich, University of Zurich, Zurich, Switzerland; 3 Centre for Water, Sanitation, Health and Appropriate Technology Development (WASHTED), Malawi University of Business and Applied Sciences (MUBAS), Private Bag 303, Blantyre 3, Chichiri, Malawi; 4 Department of Environmental Health, Malawi University of Business and Applied Sciences (MUBAS), Private Bag 303, Blantyre 3, Chichiri, Malawi

**Keywords:** Application to tertiary education, Behaviour change, Malawi, RANAS, Mental health, Physical exercise, Hunger

## Abstract

**Background:**

In Sub Saharan Africa (SSA), approximately 9 million students are enrolled in tertiary education (TE), which is 4% of the total TE enrolment globally. Barriers to higher education in SSA are numerous: poverty, food insecurity, gender, and disability, while the COVID-19 pandemic has worsened the situation. Little is known about the psychosocial factors and underlying mechanisms associated with students’ intention to apply for TE. Using a psychological theory of behaviour change, our study investigated the psychosocial and context factors associated with the application to TE.

**Methods:**

In a cross-sectional research study 821 interviews using researcher-administered questionnaires were conducted with secondary school students in rural and urban Blantyre, Malawi. A quantitative questionnaire based on the risks, attitudes, norms, abilities, and self-regulation (RANAS) model was used to assess psychosocial factors underlying application for TE. The Centre for Epidemiological Studies Depression Scale for Children (CES-DC) and household hunger scale were used to assess mental health and hunger respectively.

**Results:**

More than half of the youth were at risk to develop depression (66.5%). Girls reported experiencing more depression symptoms than boys. Around 1 in every 5 interviewed youth lived in a home experiencing moderate or severe hunger. A higher intention to apply for TE was related to perceived vulnerability, affective beliefs (joy, happiness, excitement), injunctive (approval of others) and personal norms, self-efficacy, and commitment to apply. Factual knowledge about TE application was very limited. An intention to apply for TE and self-efficacy was positively associated with regular physical exercise, but negatively associated with mental health and hunger. However, mental health moderated the effects of physical exercise on the intention to apply for TE. We found significant differences between poor and good mental health groups on intention to apply for TE in perceived vulnerability, descriptive (behaviour of others) and personal norms, self-efficacy, maintenance self-efficacy and commitment factors. The results informed a behaviour change intervention strategy to increase students’ intention to apply for TE.

**Conclusions:**

Our research findings are an important contribution to the long-term strategy of achieving the Sustainable Development Goals (SDGs) and contribute to the inclusion of vulnerable students with impaired mental health in higher education in Malawi and beyond.

**Supplementary Information:**

The online version contains supplementary material available at 10.1186/s40359-024-01562-7.

## Background

According to the World Bank [1], the supply of Sub-Saharan Africa’s (SSA) higher education has failed to improve as fast as demand across the continent. In SSA, approximately 9 million students are enrolled in Tertiary Education (TE), which is only 4% of the total number of TE students enrolled globally [2]. The Gross Enrolment Rate (GER) in Malawi is 0.8% [2] and is one of the lowest GERs in SSA and in the world. The barriers to higher education in SSA are numerous: poverty, gender, and disability [3–5]; while the COVID-19 pandemic has further worsened the situation [1, 6, 7]. However, little is known about psychosocial factors and underlying mechanisms associated with students’ intention to apply for university studies.

To identify the psychosocial factors associated with students’ intention to apply for university studies, we used the risks, attitudes, norms, abilities, and self-regulation (RANAS) approach to behaviour change [8, 9]. The RANAS model has been developed using psychological theories and consists of five psychosocial factor blocks [10–13]. Risk factors include factual knowledge, perceived vulnerability, and perceived severity of the target behaviours. Attitude factors include instrumental beliefs (beliefs about the costs and benefits) of a target behaviour and affective beliefs (feelings) arising while performing the target behaviour. Norm factors comprise perceived social influence, such as behaviour of others (descriptive norm), others’ approval (injunctive norm), and personal importance (personal norm). Ability factors include confidence in performance (self-efficacy) of a particular behaviour. Self-regulation factors cover management of conflicting goals and barriers, commitment, and remembering to perform the target behaviour. Furthermore, the RANAS model considers three domains of contextual factors: social, personal, and physical contexts. Culture, laws and policies, economic conditions, social relations, and the information environment are included in the social context. The natural and/or built environments constitute the physical context. The personal context includes age, gender, education, physical and mental health of the person, and experiencing hunger as specific condition.

The intention to apply and study at university requires effort, time, and self-efficacy. Many students do not believe that they have the capacity for academic performance and low self-efficacy beliefs, unfortunately, impede academic achievement and, in the long run, create self-fulfilling prophecies of failure and learned helplessness that can devastate psychological well-being’ [14]. Furthermore, previous research from Malawi suggests that mental health conditions such as depression can substantially impair daily activities in vulnerable students [15–18]. The prevalence of mental disorders in general population in Malawi is 29.9% [19] and depression is around 30.3% [20]. Recent research from Malawi [21] suggests that there are increasing rates of untreated mental health conditions among Malawi’s youth.

Evidence suggests that mental health may be adversely affected by food insecurity and the experience of hunger in daily life [22, 23], which may result in iron deficiency and anaemia [24], chronic health problems, and by individuals exposed to humanitarian emergencies, natural disasters, conflicts, and other kinds of violence or abuse [1].

At the same time, the health benefits of physical activity in school-aged children and youth are well established [25]. Evidence suggests a positive association between physical activity and mental health outcomes (e.g., self-esteem, anxiety, and depression) [26]. The available evidence from a recent review [27] suggests that physical activity/exercise among youth is a promising mental health intervention.

Another review [28] found evidence that there are associations among physical activity, fitness, cognition, and academic achievement in young people. Furthermore, recent research suggest that regular physical activity can improve the self-efficacy of students [29], a belief in personal capacity to execute behaviours and produce specific performance [11]. Additionally, evidence suggests that lower-self efficacy can impair academic achievement and decrease psychological well-being [14].

To investigate possibilities to improve mental health and in turn, the self-efficacy for the intention to apply and study at university among young people, factors such as physical exercise were considered. Therefore, the following four research questions were addressed: (1) Which psychosocial factors are associated with the intention to apply for TE among secondary school students? (2) Does regular physical exercise influence (a) mental health; (b) self-efficacy associated with the intention to apply for TE? and (c) the intention to apply for TE? 3) Does hunger/ food insecurity influence students’ intention to apply for TE? 4) Does mental health influence students’ intention to apply for TE?

## Methods

### Study design

This cross-sectional study included a pre-study and a quantitative survey in 20 secondary schools in Blantyre, Malawi The study participants were secondary school students. The pre-study involved key informant interviews (KII, *N* = 10) and 4 focus group discussions (FGDs) in both rural and urban areas, with secondary school girls and boys (*N* = 40). The participants for the quantitative survey were selected based on clusters (schools). Twenty secondary schools (10 from rural Blantyre and 10 from urban Blantyre) were selected randomly (using a random number generator) from a list of all secondary schools in Blantyre which was received from the Ministry of Education (i.e. Blantyre District Education office) that also provided a list of students from all the selected schools. Systematic selection of the participants (every third) from the secondary school list was applied. A priori sample size calculation was conducted for BL survey (n = [z2 * p * (1 - p) / e2] / [1 + (z2 * p * (1 - p) / (e2 * N))]) by considering the non-response rate of 20% in similar projects done in Malawi (*N* = 800). In total, 821 secondary school students were recruited for our research study. Participation in the study was completely voluntary. Data collection took place from November 2021 to March 2022.

### Questionnaires and measures

The structured, face-to-face interviews were conducted in Chichewa (the local language). The quantitative questionnaire was based on the RANAS model [9]. Most of the questions were closed, such as those about the target behaviours and the psychosocial factors (Table A1 questionnaire in Annex). Answers were measured on 5-point scale [from ‘not at all’ to ‘very much’; from ‘almost nobody’ to ‘almost all of them’]. The Center for Epidemiological Studies Depression Scale for Children (CES-DC), a 20-item screening instrument, was used to assess mental health in secondary school students [30, 31]. The CES-DC is a reliable and valid depression measurement tool (internal consistency α = 0.74–0.089; effect size = 0.72; sensitivity = 80) which consists of 20-item rating scale (from 0 to 3) with a score range from 0 to 60 (the cut-off point ≥ 15) [32]. CES-DC was validated in Rwanda [33] where researchers confirmed the usability of the tool in low-income contexts. The Malawian researcher translated the CES-DC questionnaire from English to Chichewa and another Malawian researcher retranslated it back from Chichewa to English to check the accuracy. Furthermore, the meaning of each CES-DC question was discussed with the enumerators during the training. The questionnaire was adopted to the interview situation, e.g., instead of the first person (‘I’) second person (‘You’) was used. Food security was assessed with the commonly used household hunger scale which has been validated in Malawi [34].

### Ethical, safety and regulatory issues

A team of nine local research assistants were employed to carry out face-to-face school interviews. Prior to the data collection, the interviewers attended a 3-day training for the pre-study and a 5-day training for the quantitative survey, where they were familiarized with the study, the theoretical background of the questionnaire and the questionnaire itself. The interviewers learned how to ask the different types of questions and how to fill in the questionnaire. Interviewer training also included a practice FGD and role plays. On the last day of the training, the interviewers practiced an interview at a school setting as a pre-test of the research tools.

The research protocol was approved by the Ethics Committee of the ETH Zurich in Switzerland (EK 2021-N-138) and by the ethical committee in Malawi (National Committee on Research in the Social Sciences and Humanities; NCRSH; Ref No: NCST/RTT/2/6; protocol No. P.08/21/597). Permission to visit the schools was obtained from the South-West Education Division in Blantyre. All procedures applied in the research study were in accordance with the Declaration of Helsinki. The participants were informed of the research objectives and were advised that they had the freedom to refuse participation or withdraw from the study at any time. Informed consent was obtained from all participants and their literate legal guardian. All study participants provided written informed assent. Participants were provided with a unique identifying number, and data were anonymized during data analysis. Data were accessed only by the authors.

### Statistical analysis of data

The statistical analysis of data was conducted using IBM SPSS 27 Statistics software and the PROCESS macro for SPSS. Correlations were used to investigate associations between study variables such as intention to apply for TE, mental health, hunger, physical exercise, and self-efficacy. T-tests and effect size calculations were used to compare means between poor and good mental health groups. For linear regression analysis we used intention to apply for TE as the dependent variable and the psychosocial factors of the RANAS model as independent variables. A regression analysis method, PROCESS [35] was applied to calculate moderation model. The moderation model was used to test for interaction (when two variables influence each other’s effects). Our moderation model included mental health as the moderator (M), intention to apply for TE as the outcome (Y), and physical exercise as the predictor (X). Moderation analysis was also used to test the interaction between the moderator M (mental health) and predictors X (physical exercise) in a model with outcome Y (intention to apply for TE). With evidence that X’s effect is moderated by M, the analysis should confirm X’s effect on Y at various values of the moderator (Scale: 0–60 in our model).

## Results

### The prevalence of depression

**Fig. 1:**
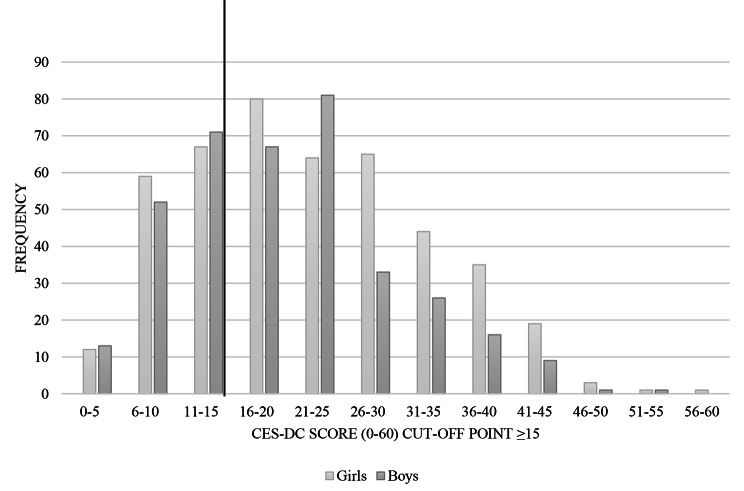
Prevalence of depression among secondary school students– comparison of girls and boys

The results revealed that prevalence of depression among secondary school students (*N* = 821) in Malawian secondary schools was 66.5% (*N* = 546) (63.1% boys, *N* = 234; 69.3% girls, *N* = 312) (cut-off point was a score of ≥ 15 (CES-DC score range 0–60)) (Fig. 1).

Correlation analysis showed a significant negative association between gender and mental health (*r*=-0.107**). Further ANOVA mean comparison analysis revealed significant differences between girls and boys (*F*(1, 821) = 10.88, *p* = 0.001). Specifically, girls (*N* = 312; *M* = 22.14 (SD = 10.72)) reported significantly more depressive symptoms than boys (*N* = 234; *M* = 19.80 (SD = 9.40)).

### Characteristics of the sample

From total 821 interviewed students, 54.8% were female (*N* = 450) and 45.2% male (*N* = 371). Study participants were on average 16.23 years old (*SD* = 1.76). The age range was between 11 and 20 years (Table 1).

**Table 1:** Sociodemographic characteristics of participants

Characteristic	*Good mental health*		*Poor mental health*		*Full sample*	
	*n*	*%*	*n*	*%*	*n*	*%*
Gender						
Female	138	50.2	312	57.1	450	54.8
Male	137	49.8	134	42.9	371	45.2
Grade						
First	77	28.0	124	22.7	201	27.3
Second	70	25.5	125	22.9	195	23.8
Third	60	21.8	135	24.7	195	23.8
Fourth	68	24.7	162	29.7	230	28.0
Setting						
Urban	136	49.5	257	47.1	393	47.9
Rural	139	50.5	289	52.9	428	52.1
School						
Government	207	75.3	409	74.9	616	75.0
Private	68	24.7	137	25.1	205	25.0
Religion						
Christians	258	93.8	507	92.9	765	93.7
Muslim	17	6.2	38	7.0	55	6.7
Other	-	-	1	0.2	1	0.1
Siblings						
Have siblings	269	97.8	525	96.2	794	96.7
Siblings at university	58	21.1	102	18.7	160	22.0
Educational level of caregiver						
No education	19	6.9	63	11.5	82	10.0
Completed primary	70	25.5	167	30.6	237	28.9
Completed secondary	98	35.6	172	31.5	270	32.9
Completed university	59	21.5	94	17.2	153	18.6
Don’t know	29	10.5	50	9.2	79	9.6

Note. *N* = 821. Participants were on average 16.23 years old (*SD* = 1.76), the age range from 11 to 20 years. Mental health: 0–1 (0 = good mental health, 1 = poor mental health, cut-off ≥ 15 with score range 0–60)

### Health related outcomes

On average, students reported that the COVID-19 emergency affected their physical or mental health ‘somewhat or rather’, affected their family’s economic situation ‘somewhat or rather’, affected their school related success (e.g., exam grades) ‘somewhat or rather’. About 40% of interviewed students reported that they missed the school in the past term because they were sick, 8.3% of students reported that they have a health-related disability, and 1.7% of students reported that they were vaccinated against COVID-19 (Table 2).

**Table 2:** COVID-19 and health related outcomes

Item	Scale	M (*SD*), % (yes)
COVID-19 impacted on physical or mental health	1–5	2.50 (1.16)
COVID-19 impacted on family economic situation	1–5	2.76 (1.24)
COVID-19 impacted on school-related success (e.g. exam grades)	1–5	2.77 (1.27)
School absence (sick past term) ^a^	Yes/No	40.2%
Health related disability ^a^	Yes/No	8.3%
Vaccination (COVID-19) ^a^	Yes/No	1.7%

Note. *N* = 821. Rating scale 1–5: 1 = not at all, 2 = somewhat, 3 = rather, 4 = quite a lot, 5 = very much. ^a^ Reflects the number and percentage of participants answering ‘yes’ to this question

### Household hunger scale (HHS)

Food security was assessed with the commonly used household hunger scale (HHS) which has been validated in Malawi [34]. As suggested by the validation study, we calculated household hunger scale from 3 items (combined score: first step (yes = 1, never = 0), second step (if yes, sometimes = 1, often = 2)) (Table 3).

**Table 3:** Hunger/food insecurity percentages

Item	0 (never)	1 (sometimes)	2 (often)
In the past 4 weeks I…			
… had no food to eat of any kind in the house	82%	15.7%	2.3%
… went to sleep at night hungry because there was not enough food	73.6%	23.8%	2.7%
… went a whole day and night without eating anything	88.3%	9.7%	1.9%

Note. *N* = 821

Further, three household hunger categories were calculated: little to no hunger in the household (score 0–1) 83.2% (*N* = 683), moderate hunger in the household (score 2–3) 14.1% (*N* = 116), and severe hunger in the household (score 4–6) 2.7% (*N* = 22). Around 16.8% of students lived in households experiencing moderate and severe hunger.

### Psychosocial factors associated with intention to apply for TE

On average, interviewed students reported that they ‘rather’ or ‘quite a lot’ intend to apply for TE (*M*=3.47 (SD = 1.19)) on a 5-point response scale from 1 (not at all) to 5 (very much). About 3.5% of students (*N* = 29) answered with ‘not at all’. The reasons why they had no intention to apply for TE were ‘no money/funding’, ‘have to help my family’, ‘don’t think I can do it’, and ‘my friends/ family don’t study either’. We found significant differences in intention to apply for TE between boys and girls (*p* = 0.000). Girls intended to apply more for TE than boys.

To investigate the behavioural determinants of intention to apply for TE we used linear regression with intention to apply for TE as the dependent variable and the RANAS psychosocial factors as independent variables. The regression analysis revealed that seven psychosocial factors are significantly associated with the intention to apply for TE: perceived vulnerability, affective beliefs, injunctive and personal norms, self-efficacy, commitment, and physical exercise. The model explained a variance of 70.5% in the intention to apply for TE. A higher intention to apply for TE was significantly related with perceived vulnerability (*β* = 0.212, *p* = 0.000), implying that for students with a stronger intention to apply for TE ‘it would be bad if they would not apply’. Affective beliefs, such as feeling good, excitement, joy, or happiness connected to the application for TE also significantly predicted intention to apply score (*β* = 0.134, *p* = 0.004), i.e., students who experienced more positive feelings are more likely to have a higher intention to apply for TE. Social norms, injunctive norms (approval/ disapproval of important other’s) (*β* = 0.108, *p* = 0.002) and personal norms (personal obligation) (*β* = 0.116, *p* = 0.004) were significant in predicting a higher intention to apply for TE as well, meaning that students with a higher intention to apply think that important people approve of their intention to pursue TE and have higher obligation to do so. The psychosocial factor self-efficacy (confidence in performance) (*β* = 0.187, *p* = 0.000) was significantly related to a higher intention to apply for TE and implies that students with stronger self-efficacy are more likely to apply for TE. Respondents’ commitment to apply for TE (*β* = 0.134, *p* = 0.001) was also significantly related to their higher intention to apply for TE (Table 4).

**Table 4:** Linear regression of RANAS psychosocial factors explaining the intention to apply for TE

Psychosocial factor	B	β	t	*p*-Value
***Risk Factors***				
Perceived vulnerability ***	0.215	0.212	5.969	0.000
Perceived severity	0.038	0.037	1.166	0.244
Factual knowledge (sum 0–6)	-0.004	-0.002	-0.092	0.926
***Attitude Factors***				
Instrumental beliefs (effort)	-0.013	-0.012	-0.601	0.548
Affective beliefs ***	0.162	0.134	2.883	0.004
***Norm Factors***				
Descriptive norm (school)	0.001	0.001	0.035	0.972
Descriptive norm (country)	-0.027	-0.019	-0.952	0.341
Injunctive norm ***	0.128	0.108	3.164	0.002
Personal norm ***	0.126	0.116	2.884	0.004
***Ability Factors***				
Action knowledge (sum 0–11)	-0.002	-0.001	− 0.0061	0.951
Skills	-0.017	-0.017	-0.838	0.402
Self-efficacy ***	0.195	0.187	4.771	0.000
Maintenance self-efficacy	-0.031	-0.026	-0.769	0.442
Recovery self-efficacy	0.049	0.044	1.530	0.126
***Self-regulation Factors***				
Action control/planning	0.020	0.018	0.503	0.615
Coping planning	-0.003	-0.012	-0.639	0.523
Remembering	-0.009	-0.007	-0.305	0.760
Commitment ***	0.141	0.134	3.395	0.001
***Additional Factors***				
Communication	0.006	0.005	0.198	0.843
Mental health	-0.044	-0.018	-0.907	0.365

Note. *p ≤ 0 0.05, **p ≤ 0.01, ***p ≤ 0.001. Adj. R^2^ = 0.705. *N* = 821; B = unstandardized beta value; *β* = standardized beta value; Behavioural question: *Do you intend to apply for university studies?* All responses were recorded on 5-point response scales with choices from ‘1 - not at all’ to ‘5– very much’. Coping plan scale: 0–1 (No/Yes); factual knowledge: sum scale (0–6); action knowledge: sum scale (0–11); Mental health: 0–1 (0 = good mental health, 1 = poor mental health, cut-off ≥ 15 with score range 0–60)

These results imply that an increase in intention to apply for TE can be expected if any of these seven significant RANAS psychosocial factors increase while all other factors hold constant. An increase in intention to apply for TE of 21.5% can be expected in students who perceive that not applying for TE is bad (perceived vulnerability). Further, an increase in intention to apply for TE should be expected from 16.2% of students who experience positive feelings, 12.8% who believe that application for TE is approved by important people, and around 12.6% in those who feel obligated to apply for TE. Additionally, an increase in intention to apply for TE of 19.5% can be expected in students who are confident that they can apply for TE and increase of 14.1% who are committed to apply for TE.

### Interaction effects between physical exercise and mental health on intention to apply for TE

To investigate whether regular physical exercise influences (a) mental health, (b) self-efficacy associated with an intention to apply for TE, and (c) the intention to apply for TE, we used correlation (Spearman and Pearson) and moderation analysis using PROCESS for SPSS 27 [35]. Our moderation model included mental health as moderator (M), intention to apply for TE as outcome (Y), and physical exercise as predictor (X).

Physical exercise was defined as ‘activities that take hard physical effort and make you breathe much harder than normal’ and we asked the question ‘do you play any sports or exercise regularly?’ (yes/no). Almost half of interviewed students reported that they play sports or exercise regularly (47.6%, *N* = 391). From those, 196 were girls (43.6%) and 195 were boys (52.6%). From those that exercise, 50.1% (*N* = 196) reported that they are running, 49.9% (*N* = 195) playing football, 20.7% (*N* = 81) dancing, 7.4% (*N* = 29) swimming, 13.8% (*N* = 54) biking, 31.5% (*N* = 123) playing netball, 0.5% (*N* = 2) practicing yoga, 7.9% (*N* = 31) weightlifting, and 21% (*N* = 82) playing other sports.

The results revealed a significant positive relationship (Spearman correlation) between (a) physical exercise and an intention to apply for university studies (*r* = 0.245**), (b) physical exercise and self-efficacy (*r* = 0.200**), and negative relationship between (c) hunger indicator and an intention to apply for TE (*r*=*-*0.111**). Further analysis showed a significant negative relationship (Pearson correlation) between mental health and the intention to apply for university studies (*r*=-0.108**), and between mental health and self-efficacy (*r*=-0.101**). Self-efficacy was measured on a response scale from 1 to 5, 1= ‘not at all’ to 5= ‘very much’ (*How confident are you that you can apply for TE?).* However, no significant correlation was found between mental health and physical exercise. In summary, an intention to apply for university studies and self-efficacy (confidence in performance) was positively associated with regular physical exercise, but negatively associated with mental health (Table 5).

**Table 5:** Correlations for study variables: physical exercise, intention to apply for TE, self-efficacy, mental health, and hunger

Variable	1	2	3	4	5
1. Physical exercise	-	0.245**	0.200**	-0.038	-0.084*
2. Intention to apply for TE			0.767**	-0.108**	*−.1*13**
3. Self-efficacy			-	-0.101*	-0.136**
4. Mental health					0.252**
5. Hunger					-

Note. *p ≤ 0.05, **p ≤ 0.01, ***p ≤ 0 0.001. *N* = 821. Physical exercising scale: 1 − 0 (Yes/no); intention to apply for university studies and self-efficacy response scale: from ‘1 - not at all’ to ‘5– very much’; metal health: sum scale (1–60)

Correlation analysis was used to determine if hunger/ food insecurity influences students’ intention to apply for university studies. The results revealed a significant negative correlation between the hunger indicator and intention to apply for TE (*N* = 821; *r=-*0.113**), between the hunger indicator and self-efficacy (*N* = 821; *r=-*0.136**), and between the hunger indicator and physical exercise (*N* = 821; *r=-*0.084*). Additionally, we found significant positive correlations between hunger and the belief that applying to TE is expensive (*N* = 821; *r =* 0.120**), and between the hunger indicator and mental health (*N* = 821; *r =* 0.252**) (Table 5).

ANOVA means comparison showed significant differences between three groups and their intention to apply for TE: little to no hunger, moderate hunger, and severe hunger in the household. In summary, students experiencing more hunger reported a lower intention to apply for TE, believed less in their self-efficacy to apply for TE, exercised less, believed more that applying for TE is expensive, and reported more symptoms of depression than students who experienced less hunger.

The intention to apply for TE correlated strongly with self-efficacy related to an intention to apply for TE (*N* = 821; *r =* 0.767**), i.e. students with a high intention to apply for TE were highly confident that they can apply for TE.

**Fig. 2:**
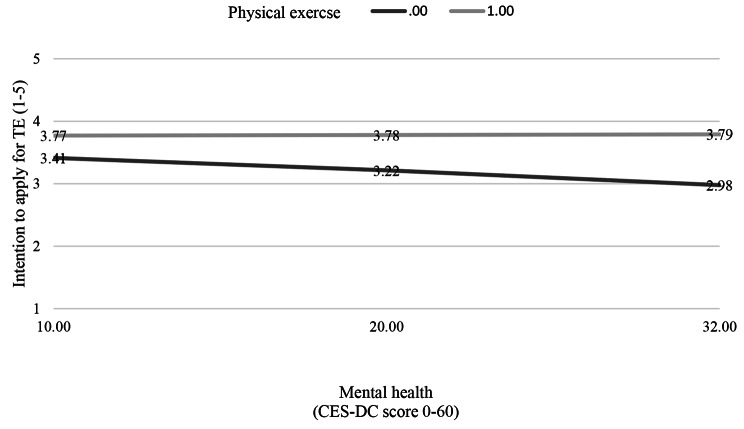
Interaction effects between mental health and physical exercise on self-reported intention to apply for TE. Mental health values are the 16th, 50th, and 84th percentiles

Further moderation analysis revealed significant interaction effects between mental health (M) and regular physical exercise (X) on the intention to apply for TE as an outcome (*b* = − 0.0207, 95% CI [0.0051, 0.0363], t = 2.60, *p* = 0.009). Mental health moderated the effects of physical exercise on the intention to apply for university studies. The relationship between physical exercise (X) and intention to apply for university studies (Y) varied as a function of the mental state of the study participants (M), meaning that the relationship depends on the mental state of students. Though the relationship was positive, it was more positive among participants with less symptoms of depression (Fig. 2).

### Mental health and the intention to apply for TE

To understand if mental health influences students’ intention to apply for TE, correlations were applied to investigate the association between mental health and intention to apply for TE. The cut-off point for mental health was a score of ≥ 15 (score range 0–60) (Fendrich et al., 1990) and coded as a dummy variable (1 − 0). The results showed significant a negative relationship (Pearson correlation) between mental health and an intention to apply for university studies (*r*=-0.108**). Additional analysis revealed significant differences (t-test for independent samples) between student groups with good vs. poor mental health in intention to apply for university studies (*p* = 0.005) and the effect size calculation (Cohen’s *d*) showed a small effect (*d =* 0.209).

Further analysis showed mean differences between students with good and poor mental health in terms of RANAS psychosocial factors underlying intention to apply for TE. Frequencies, t-test and effect-size (Cohen’s *d*) calculations were applied. The results showed significant differences in perceived vulnerability, affective beliefs (positive feelings about application for TE), social norms (descriptive norm) reflecting a belief that other learners in the school have an intention to apply for, personal norms (personal obligation to apply for TE), self-efficacy (having confidence in their performance associated with intention to apply for TE), maintenance self-efficacy (coping with barriers associated with an intention to apply for TE) and commitment to apply for TE (Table 6).

**Table 6:** Mean differences between student groups with good vs. poor mental health in psychosocial factors explaining intention to apply for TE

Psychosocial factor	Good mental health M *(SD), %, N =* 275	Poor mental health M *(SD), %, N =* 545	*p*-Value	Effect size (Cohen’s d)
***Intention to apply for TE***				
Perceived vulnerability*	3.60 (1.10)	3.39 (1.21)	0.017	0.177
***Risk Factors***				
Perceived vulnerability*	3.60 (1.10)	3.39 (1.21)	0.017	0.177
Perceived severity	3.57 (1.10)	3.41 (1.20)	*ns*	0.139
Factual knowledge (sum scale 0–6)	0.58 (0.69)	0.62 (0.72)	*ns*	-0.057
***Attitude Factors***				
Instrumental beliefs (effort)	3.22 (1.14)	3.31 (1.16)	*ns*	-0.079
Affective beliefs **	3.80 (0.86)	3.60 (1.04)	0.004	0.200
***Norm Factors***				
Descriptive norm (school) *	3.07 (1.10)	2.89 (0.93)	0.016	0.183
Descriptive norm (country)	3.28 (0.87)	3.25 (0.84)	*ns*	0.045
Injunctive norm	2.98 (1.00)	2.84 (1.01)	*ns*	0.142
Personal norm***	3.89 (0.98)	3.62 (1.14)	0.001	0.242
***Ability Factors***				
Action knowledge (sum 0–11)	0.98 (1.04)	1.01 (0.94)	*ns*	-0.041
Skills	2.36 (1.17)	2.30 (1.20)	*ns*	0.050
Self-efficacy**	3.61 (1.06)	3.38 (1.18)	0.006	0.202
Maintenance self-efficacy*	3.41 (0.92)	3.26 (1.01)	0.034	0.157
Recovery self-efficacy	3.52 (0.98)	3.39 (1.11)	*ns*	0.122
***Self-regulation Factors***				
Action control	3.61 (1.04)	3.53 (1.12)	*ns*	0.078
Remembering	3.77 (0.93)	3.66 (0.95)	*ns*	0.124
Commitment*	3.39 (1.12)	3.23 (1.18)	0.053	0.143
***Additional Factors***				
Communication	2.36 (1.11)	2.34 (1.14)	*ns*	0.014

Note. *p ≤ 0.05, **p ≤ 0.01, ***p ≤ 0.001. *N* = 821. Behavioural question: *‘Do you intend to apply to university studies?’* All responses were recorded on 5-point response scales with choices from ‘1 - not at all’ to ‘5– very much’. Coping plan scale: 0–1 (No/Yes); factual knowledge: sum scale (0–6); action knowledge: sum scale (0–11); Mental health: 0–1 (0 = good mental health, 1 = poor mental health, cut-off ≥ 15 with score range 0–60). Cohen’s *d*, small: *d =.*20, medium: *d =.*50, large: *d* = 0.80

## Discussion

### Interpretation of results

This study investigated psychosocial factors and underlying mechanisms, such as mental health, hunger, and physical exercise, associated with students’ intention to apply for TE. The overall aim of the study was to develop Behaviour Change (BC) intervention strategies to increase the intention to apply for TE among Malawian secondary school students.

More than half of the students assessed in our study were at risk to develop depression (66.5%), which is in line with the findings from previous study in peri-urban locations [15]. Females reported experiencing more depression symptoms than males. Interviewed students reported that the COVID-19 emergency affected their physical or mental health on average somewhat or rather, meaning that other factors influenced students’ mental health as well.

On average, interviewed students reported that they ‘rather’ or ‘quite a lot’ intend to apply for TE. The most important determinants of intention to apply for university studies were perceived vulnerability about how bad it would be if they do not apply, affective beliefs (positive feelings) associated with intention to apply, social norms (injunctive norm, approval of important people associated with intention to apply) and personal norm (personal obligation to apply for TE), self-efficacy which reflects confidence in performance, and commitment to apply. Most students had little factual knowledge about the application process. In summary, students with a stronger intention to apply for TE perceive themselves more vulnerable if they do not apply, experience more positive feelings connected to the application process, are more likely to think that important people approve of their intention to pursue TE, feel more morally obligated to apply, have higher self-efficacy, and are more committed to apply. However, independent of students’ intention strength, their factual knowledge about the application process was low. Consequently, by targeting those psychosocial factors with BC interventions we expect higher intention to apply for TE among students after the BC intervention.

In terms of physical exercise, almost half of interviewed students reported that they play sports or exercise regularly. Importantly, the associations of physical exercise with the intention to apply for TE, and of physical exercise with self-efficacy (confidence in performance) were positive. However, no direct association was found between mental health and physical exercise. We assume that the relationship between physical exercise and mental health could be moderated by frequency and intensity of physical exercise, which was not assessed in our survey. However, we detected a positive association between physical exercise and an intention to apply for university studies. Mental health moderated the effects of physical exercise on the intention to apply for TE. In summary, though the relationship was positive, it was more positive among participants with fewer symptoms of depression. Our findings are in line with previous research that participation in physical activity can improve self-efficacy in young people [29]. Impaired mental health negatively influenced the association between physical activity and the intention to apply which confirms the results from previous research about connection of mental health and physical exercising [26].

Mental health influenced students’ intention to apply for university studies directly. Students who were at risk of depression expressed low intention to apply for university studies compared to the non-depressed students’ group. We found differences in several psychosocial factors explaining intention to apply for university studies between students with good vs. poor mental health. Perceived vulnerability, affective beliefs (positive feelings), descriptive norms (behaviour of other learners at school), self-efficacy (confidence in performance), maintenance self-efficacy (coping with barriers) and commitment were higher in the group of students with good mental health compared to those with poor mental health. Students with good mental health were more likely to believe that if they do not apply for TE it would be bad for them. They also experienced more positive feelings associated with studies at university, they think that other students in school will apply for TE, they can better cope with barriers related to the application process and are more committed to apply for TE compared to those with impaired mental health. Our findings confirmed results from previous research from Malawi, which suggests that internal mental health conditions such as poor mental health and depression can substantially impair daily activities in vulnerable people [1, 15–18]. Previous research suggest that lower-self efficacy can impair academic achievement and decrease psychological well-being [14].

Our study results suggest that around 1 in every 5 interviewed students lived in households experiencing moderate or severe hunger. Students who experienced more hunger reported lower intention to apply for TE, believed less in their self-efficacy to apply for TE, exercised less, and reported more symptoms of depression than students who experienced less hunger. These findings are in line with evidence that mental health can be adversely affected by food insecurity and the experience of hunger in daily life [22, 23]. Our results indicate that students who experienced more hunger in their daily life believed that applying for TE is expensive; we assume that they are living in poorer households, which could explain their lower intention to pursue TE because of lower income compared to children who have no experience of hunger.

In summary, our study results revealed the direct and indirect associations between mental health (depression), the state of hunger, regular physical exercise, and the intention to apply for university studies and confirmed findings from previous research. Ultimately, these results will be used to develop evidence-based BC intervention strategies aimed at increasing the application rate for university studies among Malawian students while taking into account the important role of mental health. Our research helps to address current bottlenecks in the achievement of the Sustainable Development Goals and will help to further the inclusion of vulnerable students with impaired mental health in higher education.

### Limitations

The CES-DC assessment tool has been validated in Rwanda [33], but not in Malawi. However, we have previously used the CES-DC in Malawian schools in a project with the Malawian Red Cross (*N* = 400, results unpublished) and it showed similar results as in this study. These results are derived from a limited set of schools that are near the commercial capital of Blantyre and are not necessarily representative of students who attend schools in other parts of Malawi.

### Practical implications

#### Intervention strategy for intention to apply for TE

The study results revealed that an intervention strategy should target the following psychosocial factors aiming to increase intention to apply for TE: perceived vulnerability, affective beliefs (positive feelings), social norms (injunctive and personal), self-efficacy (confidence in performance) and commitment. As the knowledge about the application process was very limited, the factual knowledge should be targeted with an information-based intervention as well. Additionally, our study results suggest targeting physical exercise and mental health. Behaviour change techniques (BCTs) presented in Table A2 (see Annex) are selected from the RANAS BCT’s catalogue (www.ranasmosler.com). An intervention strategy will be discussed during the intervention development workshop and a final intervention implementation guide will be developed.

## Conclusions

Our research findings are an important contribution to the long-term strategy of achieving the Sustainable Development Goals and contribute to the inclusion of vulnerable students with impaired mental health in higher education. The output of our research includes program designs, interventions, and best practices; the documented effectiveness and cost-effectiveness from future evaluation results will assist with the scale-up within Malawi and beyond. The impact of these interventions will lead to better mental health among students, more available data for decision-makers, increased representation of women within academia, and improved access to higher paying careers for graduates.

The ultimate outcome of this work is that mental health should be recognized by educators and education institutes as a key determinant for success, and that an increased number of highly skilled African graduates will be able to design and implement the national strategies required to meet the SDGs (SDG 3: good health and wellbeing; SDG4: quality education; SDG5: gender equality; and SDG 10: reduced inequalities) without relying on external experts and therefore foster a sense of national pride and self-sufficiency. This is the first comprehensive body of work to apply theories from social psychology to issues related to enrolment on the African continent, as a means of holistically addressing the deficit of skilled workers required for self-directed development.

### Electronic supplementary material

Below is the link to the electronic supplementary material.

Supplementary Material 1

## Data Availability

The dataset generated and analysed during the current study are available in the [https://osf.io] repository, [https://osf.io/x46c7/?view_only=8fa576c62b1345c0a5fde64b8405437a]
