# Supplementary material for: Psychosocial factors associated with intention to pursue tertiary education among Malawian students: the moderating effect of mental health
Source: BMC Psychol. 2024 Feb 9;12:65. doi: 10.1186/s40359-024-01562-7 (PMC10854067; doi:10.1186/s40359-024-01562-7)
Supplement: Supplementary file 1 — Supplementary Material 1 [file 40359_2024_1562_MOESM1_ESM.docx]

**Annex**

Table A1 Questionnaire on the RANAS psychosocial factors explaining intention to apply for TE

| **Behaviour Determinants** | **Selected Items** |
| --- | --- |
| **Risk Factors** | |
| Perceived vulnerability | How bad would it be for you if you do not apply for the University studies? |
| Perceived severity | If you will not apply or your application will be rejected, how severe would it impact your life? |
| Factual knowledge | Do you know how to apply to university studies?  Please tell me what kind of documents you need if you want to apply for university studies? |
| **Attitudinal Factors** | |
| Instrumental beliefs | Does applying to university studies take a lot of time?  Is it effortful for you to apply for university studies?  Is it expensive for you to apply for university studies? |
| Affective beliefs | Would you feel happy if you applied to university?  Would you feel good if you applied to university?  Would you feel excited if you applied to university?  Would you feel anxious if you applied to university?  Would you enjoy studying at university?  Would it make you happy to study at university? |
| **Normative Factors** | |
| Descriptive norm (school) | In your opinion, how many other learners in your school will apply and study at university? |
| Descriptive norm (country) | In your opinion, how many other learners in Malawi will apply for university studies? |
| Injunctive norm | Do your teachers think you have to apply for university studies?  Do your friends think you have to apply for university studies?  Do your caregivers think you have to apply for university studies?  How much do people who are important to you approve your intention to study at university?  How much do famous people approve of your intention to study at university? |
| Personal norm | Is it important to you to apply for university studies? |
| **Ability Factors** | |
| Action knowledge | What do you need to apply to university studies? |
| Skills | Does it require to have skills for you to apply for university studies? |
| Self-efficacy | How confident are you that you can apply to university studies? |
| Maintenance self-efficacy | Imagine that the application system has crashed. How sure are you that in this situation, you will be able to apply before the deadline?  Imagine you need to apply to university, but your friends are waiting for you. They will not wait long. You are in a hurry! How sure are you that in this situation, you will work on your application to meet a deadline? |
| Recovery self-efficacy | Imagine that you were sick and was not able to apply for university studies. How sure are you that in this situation, you will try to apply next year?  How confident are you that you can continue applying for university studies even if you don't have the application fee? |
| **Self-Regulation Factors** | |
| Action control | Will you pay attention to applying at a time before the deadline? |
| Coping planning | Do you have a plan when, where, and how to apply for university studies? If yes, what kind of plan? |
| Remembering | Do you always remember to meet deadlines with your schoolwork? |
| Commitment | Would you feel guilty if you don't apply for university studies?  Are you committed to applying for university studies? |
| **Additional Factor** | |
| Communication | How often do you talk to others about application to university studies? |
| **Behaviour**  Intention to apply for TE | Do you intend to apply for university studies? |

*Notes*. Response scales: 5-point scale for all RANAS psychosocial factors and intention to apply [from ‘not at all’ to ‘very much’; from ‘at no time’ to ‘almost each time’; from ‘never’ to ‘very often’; from ‘nobody’ to ‘almost all of them’], [yes; no; I don’t know]. Factual knowledge sum scale ranged from min. 0 to max. 6 (yes/no questions). Action knowledge sum scale ranged from min.0 to max. 11 (yes/no questions).

Table A2 Intervention strategy for intention to apply for TE

| Targeted psychosocial factor and result  Students whose intention to apply for TE is lower… | Behaviour change technique (BCT) | Activities and  messages | Communication  channel |
| --- | --- | --- | --- |
| **Perceived vulnerability**  … perceive less that not applying to TE would be bad for them | BCT 3: Inform about and assess personal risk | Present qualitative and quantitative assessments individually for each person in such a way that the person realizes about advantages of applying to university studies | Counselling services, school or group meetings, inviting students |
| **Factual knowledge**  … know less about the possibilities and process of application to TE | BCT 1: Present facts | Present information about the circumstances and possibilities of application to TE | Posters, flyers, school meetings, counselling services |
| **Affective beliefs (feeling good, happy, excited)**  … feel less good, happy, and excited when they think about applying to TE  **Affective beliefs (happy, enjoy)**  … feel less happy and enjoy less when they think about studying at university | BCT 8: Describe feelings about performing and about consequences of the behaviour | Present the performance and the consequences of a studying at university as pleasant and joyful and its omission. The participant can be invited to assess and describe his/her feelings by him/herself, others can testify their feelings or feelings can be induced. | School meetings, group meetings, video spots, posters with happy graduates |
| **Descriptive norm (others behaviour at school)**  … think that only some leaners from their school will apply for university studies | BCT 9: Inform about others’ behaviour  BCT 10: Prompt public commitment | Point out that a desired behaviour/intention is already adapted by other learners.  Let students commit to a favourable behaviour or intention and make their commitment public, thus showing to others that there are students who applying to TE and graduate as well. The commitment can be oral in front of an audience, written at a public place, or by means of a sign attached to the committed person so that others can see it. | School meetings, group meetings, video spots, invite graduates or students |
| **Injunctive norm (others approval)**  … think that important people like caregivers, teachers, important and famous people only somewhat approve application to university studies | BCT 11: Inform about others’ approval / disapproval | Point out that important people support the desired behaviour or intention (e.g. studying at university). Important people may be family members, popular persons etc. E.g. convince an important person to state in front of the students that application to TE is important, is easy to fill in, and studying is beneficial for students, the whole community and country. | Video spots, TV spot, school meetings |
| **Personal norm**  …feel only somewhat important to apply for university studies | BCT 13: Provide a positive group identity  BCT 14: Prompt identification as role model | Describe students already engaged in the behaviour in an attractive way, e.g. as modern and up-to-date, to view the proposed behaviour change as having a positive influence on one’s identity. Youth may aspire to be one of those positively described persons and therefore change behaviour/intention.  Ask university students or students who intend to study at university to set a good example by engaging in the desired behaviour so as to influence others’ behaviours by one’s own behaviour. | Posters with happy university students, inviting students from university |
| **Self-efficacy (confidence in performance)**  … feel only somewhat confident that they can apply to university studies | BCT 16: Provide infrastructure  BCT 21: Organize social support | Demonstrate how easy an application to TE can be fill-in. In a group of interested students (max 5 people) an instructor shows step by step how to fill in application form. The instructor also provides the document which includes all steps of application process*.*  Prompt youth to seek practical or emotional support from friends or relatives, counselling services and/or to initiate social support groups. This can include monetary and physical help, action knowledge or (verbal) emotional support. | Counselling services, group meetings |
| **Maintenance self-efficacy (coping with barriers)**  … feel only somewhat that they can overcome barriers regarding application for university studies | BCT 24: Reattribute past successes and failures | Prompt youth to attribute successes as personal achievements and failures to adverse circumstances or to not yet developed but achievable skills instead of to personal deficiency. | Counselling services, group meetings |
| **Commitment**  … feel only somewhat committed to apply for university studies | BCT 35: Prompt goal setting | Invite participants to formulate a behavioural goal or intention (e.g. I will pay attention to apply for TE before deadline) | Counselling services, group meetings |
| **Physical exercise**  …exercising less regularly | BCT 1: Present facts | Present information about the advantages of regular physical exercise, present physical exercising possibilities | Sport competitions, video-spots, posters |
| **Mental health** | BCT 21: Organize social support | Prompt youth to seek practical or emotional support from friends or relatives, counselling services and/or to initiate social support groups. This can include monetary and physical help, action knowledge or (verbal) emotional support. | Counselling services, group meetings, group meditation practices |
